# Supplementary material for: CCHamide-2 Signaling Regulates Food Intake and Metabolism in Gryllus bimaculatus
Source: Insects. 2022 Mar 25;13(4):324. doi: 10.3390/insects13040324 (PMC9026500; doi:10.3390/insects13040324)
Supplement: Supplementary file 1 [file insects-13-00324-s001.zip › insects-1625740-supplementary.pdf]

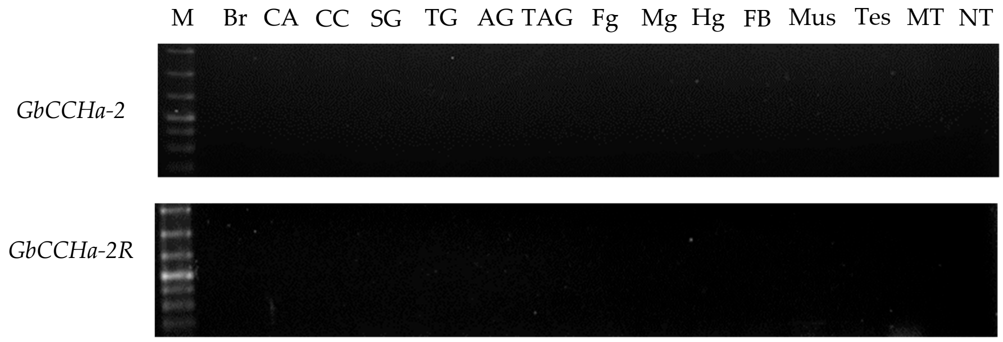

Figure S1. No-RT negative control and no-template control of RT-PCR. Br, brain; CA, corpora allata; CC, corpora cardiaca; SG, subesophageal ganglion; TG, thoracic ganglia; AG, abdominal ganglia; TAG, terminal abdominal ganglion; Fg, foregut; Mg, midgut; Hg, hindgut; FB, fat body; Mus, muscle; Tes, testis; MT, Malpighian tubules. M, marker. NT, no-template control.
